# Circulatory Disturbances in Acute Coronary Syndrome Patients Undergoing Percutaneous Coronary Intervention: Mechanisms, Management, and Outcomes

**DOI:** 10.3390/jcm14207250

**Published:** 2025-10-14

**Authors:** Tarek Abdeldayem, Ashan Gunarathne, Mohamed Farag, Mohammad Alkhalil, Mohaned Egred

**Affiliations:** 1 Cardiothoracic Centre, Freeman Hospital, Newcastle-upon-Tyne NE7 7DN, UK; mohamedfarag@nhs.net (M.F.); mohammad.alkhalil@nhs.net (M.A.); m.egred@nhs.net (M.E.); 2 Nottingham University Hospitals, Nottingham NG5 1PB, UK; a.gunarathne@nhs.net

**Keywords:** cardiogenic shock, acute coronary syndrome, mechanical circulatory support

## Abstract

Circulatory disturbances in patients with acute coronary syndrome (ACS) undergoing percutaneous coronary intervention (PCI) present significant challenges in interventional cardiology. This review examines the pathophysiological mechanisms, management strategies, and outcomes associated with these hemodynamic complications, ranging from transient hypotension to severe cardiogenic shock (CS). The complex interplay between myocardial ischemia, reperfusion injury, and procedural stress creates a dynamic circulatory environment that requires careful monitoring and intervention. The review analyzes various causes of circulatory disturbances, including vasovagal reflexes, allergic reactions, cardiac arrhythmias, acute ischemia, and procedural complications. It emphasizes the importance of early recognition and appropriate management of these conditions to improve patient outcomes. The progression from hypotension to CS is examined, with a focus on assessment tools, prognostication, and revascularization strategies. The role of mechanical circulatory support devices in managing severe circulatory compromise is discussed, including intra-aortic balloon pumps, Impella devices, and veno-arterial extracorporeal membrane oxygenation (VA-ECMO). Recent randomized controlled trials have yielded mixed results regarding the efficacy of these devices, highlighting the need for a nuanced, patient-centered approach to their use. This comprehensive analysis provides clinicians with a framework for anticipating, identifying, and managing circulatory disturbances in ACS patients undergoing PCI. It underscores the importance of risk stratification, multidisciplinary approaches, and ongoing research to optimize patient care and improve outcomes in this high-risk population.

## 1. Background

Circulatory disturbances in patients with acute coronary syndrome (ACS) undergoing percutaneous coronary intervention (PCI) represent a significant challenge in interventional cardiology. These hemodynamic complications, ranging from transient hypotension to severe cardiogenic shock (CS), can profoundly impact patient outcomes and procedural success [1,2]. The complex interplay between myocardial ischemia, reperfusion injury, and the physiological stress of the intervention itself creates a dynamic and potentially precarious circulatory environment [3,4,5].

This review article aims to provide a comprehensive analysis of the circulatory disturbances encountered in ACS patients during PCI, with a particular focus on hypotension, pre-shock states, and CS. We will examine the pathophysiological mechanisms underlying these complications, including blood pressure fluctuations, alterations in tissue perfusion, and signs of end-organ hypoperfusion. By characterizing the progression of circulatory instability in these patients, we seek to enhance understanding of the various stages of hemodynamic compromise and their clinical implications.

The timing of circulatory disturbances in relation to PCI is of particular interest, as these complications can manifest before, during, or after the procedure [4]. Each temporal phase presents unique challenges in terms of etiology, diagnosis, and management [5,6]. The multifactorial nature of these disturbances, which can include vagal responses, myocardial stunning, and procedure-related complications such as coronary perforation or no-reflow phenomenon, necessitates a nuanced approach to patient care [4].

By synthesizing current evidence and expert opinion, this review aims to provide clinicians with a comprehensive framework for anticipating, identifying, and managing circulatory disturbances in ACS patients undergoing PCI. Through this analysis, we hope to contribute to improved patient outcomes and advance the field of interventional cardiology in managing this high-risk patient population.

This review was conducted using a structured literature search of PubMed, EMBASE, and Google Scholar. Search terms included “acute coronary syndrome,” “hypotension,” “cardiogenic shock,” “mechanical circulatory support,” “Impella,” “IABP,” “ECMO,” “no-reflow,” and related keywords. Priority was given to randomized controlled trials (RCTs), large registries, and meta-analyses, while narrative reviews and expert consensus documents were included to provide contextual interpretation.

Studies were included if they involved adult patients undergoing PCI for ACS with reported hemodynamic complications or MCS use. Exclusion criteria were case reports, pediatric studies, and non-English publications.

## 2. Hypotension

Hypotension during and after PCI represents a multifaceted clinical challenge that demands careful consideration and prompt intervention. This hemodynamic instability, characterized by a significant decrease in blood pressure, can arise from various physiological and procedural factors associated with the intervention [7]. The complexity of this condition lies in its potential to rapidly deteriorate into CS, a life-threatening complication with high morbidity and mortality rates [6,7]. Recognizing the early signs of hypotension and implementing appropriate therapeutic strategies are crucial steps in preventing adverse outcomes and optimizing patient care in the context of PCI procedures. The following discussion aims to explore the etiology, risk factors, and management strategies for hypotension in the peri-PCI period, emphasizing the importance of vigilant monitoring and timely intervention to improve patient outcomes.

### 2.1. Vasovagal Reflexes

The activation of vagal reflexes can transiently reduce cardiac output. A notable instance is the Bezold–Jarisch reflex (BJR), commonly observed in inferior myocardial infarction (MI) during reperfusion. BJR is characterized by a triad of bradycardia, hypotension, and peripheral vasodilation, which is triggered by the stimulation of cardiac vagal afferents, predominantly located in the inferoposterior wall, during ischemia or sudden reperfusion [8]. The reflex induces an abrupt parasympathetic surge and sympathetic withdrawal; even when heart rate is maintained through pacing, significant vasodilation occurs, thereby reducing both preload and afterload. Clinically, this phenomenon can manifest during a right coronary artery (RCA) or left circumflex artery (LCx) PCI for inferior ST-elevation myocardial infarction (STEMI), resulting in sudden hypotension and often bradycardia immediately following the reopening of the artery [8,9]. Management is supportive, involving intravenous fluids, atropine, and vasoconstrictors such as epinephrine or phenylephrine, until the reflex subsides. Additionally, a more routine vasovagal reaction, unrelated to reperfusion, may occur due to pain or anxiety, similarly causing transient hypotension and bradycardia through increased vagal tone [10].

### 2.2. Allergic/Anaphylactoid Reaction

A sudden response to contrast media or other substances (such as protamine) can result in widespread vasodilation, capillary leakage, and shock. Although uncommon, anaphylaxis in the catheterization lab manifests as severe low blood pressure, often accompanied by rapid heartbeat, skin rash, or bronchospasm [11]. This reaction can occur within minutes following the injection of contrast. Immediate intervention is necessary, involving the use of epinephrine, steroids, and fluids. In one instance, contrast-induced anaphylaxis was considered among the possible causes of abrupt hypotension during PCI (Importantly, the patient’s low blood pressure did not respond to steroids or antihistamines, suggesting that an allergy was unlikely [12].

### 2.3. Cardiac Arrhythmias

Both bradyarrhythmias and tachyarrhythmias can precipitate hypotension during PCI. For instance, transient high-grade AV block or sinus pauses (especially during RCA/inferior wall ischemia) will drop cardiac output and blood pressure [13]. On the other end, a ventricular tachycardia (VT) or rapid atrial arrhythmia can compromise filling time and output. These arrhythmias may be procedure-related (e.g., ischemia-induced or triggered by guidewire irritation) and need prompt management if they cause hemodynamic instability [14].

### 2.4. Acute Ischemia, No-Reflow and Myocardial Stunning

Hypotension may indicate insufficient myocardial perfusion during intervention. The no-reflow phenomenon is characterized by impaired microvascular perfusion despite the patency of the epicardial artery. This condition is most frequently observed during PCI for acute myocardial infarction (MI) or in saphenous vein graft interventions. Mechanistically, no-reflow results from microvascular obstruction due to distal microthromboembolism, endothelial swelling, inflammatory damage, and edema, leading to persistent ischemia [15]. Clinically, no-reflow often manifests as sudden hypotension, bradycardia, and ST-segment changes, accompanied by recurrent chest pain, even after successful stent placement. One registry reported no-reflow in approximately 2.3% of MI PCI cases, while smaller studies suggest a higher incidence, ranging from 10% to 40% in high-risk MI cases. Notably, no-reflow is a significant cause of severe intraprocedural hypotension; one study identified it as the cause in 83% of cases of profound hypotension during PCI that required intracoronary epinephrine administration [16,17]. The treatment of no-reflow involves the use of intracoronary vasodilators, such as adenosine, verapamil, and nitroprusside, along with supportive care to enhance coronary microcirculation. Persistent no-reflow indicates a large area of myocardial stunning, often signaling the onset of CS. Additionally, myocardial stunning is a transient, reversible depression of left-ventricular contractility that persists after the relief of acute ischemia, despite viable myocardium and restored epicardial patency; classically, wall-motion abnormalities may take hours to days to normalize after reperfusion [18]. This phenomenon is characterized by a prolonged period of reduced contractility even after blood flow has been restored. During PCI, the temporary occlusion of a coronary artery can lead to this stunning effect. The stunned myocardium, while still viable, exhibits impaired function, which can contribute to a temporary decrease in cardiac output. This reduction in cardiac performance may result in hypotension following the procedure. The exact mechanisms of stunning are complex, but they likely involve calcium overload within the cardiomyocytes and increased production of reactive oxygen species during reperfusion. These factors can interfere with the normal contractile function of the heart muscle cells. The hypotension that follows is a consequence of this reduced contractility and the subsequent decrease in cardiac output. It is important to note that while stunning is generally reversible, it can take hours or even days for the myocardium to fully recover its function after the ischemic event [19,20].

A related but distinct ischemic complication is right ventricular infarction (RVI), which most frequently occurs in the context of inferior STEMI. RVI results in disproportionate hypotension despite a preserved left ventricular ejection fraction, highlighting the right ventricle’s sensitivity to ischemia and its reliance on adequate preload. In contrast to left ventricular dysfunction, hypotension in RVI is aggravated by the administration of nitrates or excessive diuresis, whereas cautious fluid resuscitation to enhance venous return is generally advantageous. The definitive treatment remains the urgent reperfusion of the culprit vessel, and temporary pacing may be necessary in the presence of bradyarrhythmia. Recognizing this unique hemodynamic profile is essential, as tailored management can swiftly reverse shock physiology and prevent progression to CS [7,21].

### 2.5. Procedural Complications

Procedural mechanical complications during PC can lead to sudden circulatory collapse by acutely impairing cardiac filling or forward flow. A quintessential example is cardiac tamponade resulting from coronary or chamber perforation, where rapid accumulation of pericardial blood elevates intrapericardial pressure, restricts diastolic filling, and causes a sharp decline in stroke volume and arterial pressure [22]. Any unexplained intra-procedural hypotension should prompt immediate evaluation for tamponade—preferably with point-of-care echocardiography—as timely pericardiocentesis is often lifesaving. Notably, delayed tamponade may occur when a small perforation or wire-related injury results in a slower pericardial effusion that becomes clinically evident during recovery; new hypotension in the hours following PCI warrants urgent reassessment even if the procedure appeared uncomplicated [23]. Clinical findings such as elevated jugular venous pressure, muffled heart sounds, and pulsus paradoxus support the diagnosis. Beyond overt perforation, intramural hematoma or a loculated pericardial clot can create “dry” or pseudo-tamponade with chamber compression and similar hemodynamic consequences without a large free effusion [24,25].

An additional critical mechanism is the damping or ventricularization of the pressure waveform, which transpires when the guiding catheter is inserted excessively into the coronary ostium. This can precipitate significant hemodynamic disturbances, resulting in hypotension and ischemia, particularly in the presence of ostial disease. This issue is notably more severe in left system interventions, potentially causing extensive ischemia and rapid clinical deterioration. Key indicators of this complication include a sudden alteration in the arterial pressure waveform, the absence of the dicrotic notch, and ventricularization of the tracing. Prompt repositioning of the catheter is essential for management, while prevention necessitates appropriate catheter selection, meticulous engagement techniques, and continuous monitoring of pressure waveforms. This complication underscores the necessity of precise technique and vigilant hemodynamic monitoring during PCI, especially in patients with unstable hemodynamics or complex anatomy [26].

In addition to mechanical injury, it is imperative to consider bleeding complications, particularly in the period following PCI. Retroperitoneal hemorrhage is a well-documented cause of post-procedural hypotension, whereas gastrointestinal bleeding, associated with the concurrent use of dual antiplatelet therapy and parenteral anticoagulation, may initially be occult. In such instances, hypotension may precede overt clinical manifestations such as melena or hematemesis, highlighting the necessity for vigilance in patients who exhibit unexplained hemodynamic instability after PCI [7].

Other catastrophic but rare iatrogenic events—including catheter-induced aortic root dissection—may similarly trigger acute hemodynamic collapse and require expedited definitive management (e.g., surgical repair). Prompt recognition of these entities and execution of a structured response pathway—hemodynamic stabilization, immediate imaging, and targeted intervention—are critical to restoring perfusion and preventing progression to CS [27].

## 3. Cardiogenic Shock

CS represents the extreme end of circulatory disturbance in patients with ACS. It is a critical complication characterized by severe impairment of cardiac function, leading to inadequate tissue perfusion and organ dysfunction. Despite advances in treatment strategies, CS continues to be associated with unacceptably high mortality rates of 40–50% in ACS patients [28,29].

CS is defined by critical end-organ hypoperfusion and hypoxia resulting from reduced cardiac output due to primary cardiac disorders. Clinically, it manifests as persistent hypoperfusion unresponsive to volume replacement, evidenced by cold extremities, oliguria, altered mental status, and elevated arterial lactate levels [30]. The most common cause, accounting for approximately 80% of cases, is left or right ventricular failure following acute myocardial infarction (AMI). Less frequent causes include mechanical complications of AMI such as ventricular septal rupture, free wall rupture, and acute severe mitral regurgitation [30,31].

### 3.1. Assessment and Prognostication

Early identification of patients at risk of developing CS is crucial for improving outcomes. Various biomarkers and risk scores have been developed to aid in this process. Arterial lactate, particularly the 8 h value, has been shown to be a strong predictor of mortality [30,31]. The IABP-SHOCK II score, which incorporates biomarkers such as lactate, creatinine, and glucose, has been validated for risk stratification in AMI-CS patients. Early recognition allows for timely intervention and appropriate management strategies, potentially reducing the progression to full CS and improving patient outcomes [32,33].

Invasive assessment serves as a vital tool. While not essential for initial diagnosis in everyday clinical scenarios, the measurement of specific hemodynamic parameters through invasive monitoring provides critical data for confirming the diagnosis and directing treatment strategies. This typically involves evaluating the cardiac index, which is often decreased, and the pulmonary capillary wedge pressure, which is usually elevated in CS [4,30].

The Swan-Ganz catheter, a type of pulmonary artery catheter, is frequently employed for this purpose. It enables the measurement of crucial variables including cardiac output, pulmonary artery pressure, and mixed venous oxygen saturation. A relatively new metric, the pulmonary artery pulsatility index (PAPI), has shown particular promise in evaluating right ventricular function in CS patients [33].

These invasive assessments are particularly valuable in cases where patients show unexpected responses to initial treatments. They offer detailed insights into the condition of both the right heart and the systemic circulation. Such information is invaluable for customizing treatment approaches and closely monitoring how patients respond to various interventions. Ultimately, this comprehensive invasive assessment can significantly contribute to improving outcomes in this critically ill patient population [3,30].

To standardize the assessment and management of CS, the Society for Cardiovascular Angiography and Interventions (SCAI) has developed a classification system comprising five stages: A (At risk), B (Beginning), C (Classic), D (Deteriorating), and E (Extremis), (Table 1). This classification aims to provide a framework for assessing the severity of CS and guiding treatment decisions. Stage A includes patients at risk but not yet experiencing shock, while Stage E represents extreme shock with circulatory collapse requiring ongoing CPR or multiple pressors or mechanical circulatory support devices [4].

The SCAI classification has been validated in several retrospective cohorts and one prospective study, demonstrating good correlation with prognosis. However, it is important to note that while this classification system is useful for evaluating the course of CS over time, it is currently not well-suited as an immediate numerical score for decision-making in the catheterization laboratory.

### 3.2. Coronary Revascularization

Timely management of ongoing ischemia is crucial in the treatment of CS complicating acute myocardial infarction (AMI). Coronary revascularization stands at the forefront of this management strategy, with a substantial body of evidence supporting its efficacy [30].

The landmark SHOCK trial [35], published in 1999, laid the foundation for early revascularization in CS. While it did not show a significant difference in 30-day mortality (46.7% vs. 56.0%, *p* = 0.11), it demonstrated a marked improvement in one-year survival (46.7% vs. 33.6%, *p* = 0.025) for early revascularization compared to initial medical stabilization. Long-term follow-up at 6 years further confirmed this benefit (32.8% vs. 19.6%, *p* = 0.03) [36].

Subsequent registry studies have reinforced the importance of timely intervention. The FITT-STEMI trial [37], involving 12,675 patients, showed that every 10 min delay in treatment was associated with 3.31 additional deaths per 100 PCI-treated CS patients, highlighting the critical nature of rapid revascularization.

The CULPRIT-SHOCK trial [38,39], published in 2017, provided crucial insights into the optimal revascularization strategy for CS patients with multivessel disease. This randomized study of 706 patients demonstrated that culprit-lesion-only PCI resulted in lower 30-day mortality compared to immediate multivessel PCI (43.3% vs. 51.6%, *p* = 0.03). The one-year follow-up confirmed this benefit, with mortality rates of 50.0% vs. 56.9% (*p* = 0.048) favoring the culprit-lesion-only approach.

Even in older populations (≥75 years) with CS, early revascularization appears to offer significant benefits according to an observational study of 111,901 patients in the United States from 1999 to 2013 [40]. The study found that the utilization of PCI in older adults with STEMI and CS increased from 27% in 1999 to 56% in 2013, accompanied by a substantial reduction in in-hospital mortality rates from 64% to 46% over the same period. Using propensity score matching methods to account for potential confounders, PCI was associated with a lower risk of in-hospital mortality across all quintiles of propensity score (Mantel-Haenszel OR: 0.48, 95% CI 0.45–0.51). This reduction in mortality risk was consistent across all four United States Census Bureau regions. The study’s findings suggest that, despite the higher risks associated with advanced age and CS, early revascularization with PCI can significantly improve survival outcomes in this vulnerable population.

The current body of evidence strongly supports early revascularization in CS, with a preference for culprit-lesion-only PCI in multivessel disease, followed by staged revascularization after clinical stabilization, (Table 2). This approach often leaves a high residual SYNTAX score, and the potential advantage of immediate surgical revascularization remains unclear [41]. Smilowitz et al. [42] conducted a large retrospective study that compared CABG versus PCI in patients with MI complicated by CS. Among 386,811 hospitalizations for MI with CS, 62.4% underwent revascularization, with PCI in 44.9% and CABG in 14.1% of cases. CABG was associated with significantly lower in-hospital mortality compared to PCI. In the overall population, CABG (without PCI) had a mortality rate of 18.9% versus 29.0% for PCI alone (*p* < 0.001). This mortality benefit persisted in a propensity-matched analysis of 19,882 patients, where CABG was associated with 19.0% mortality compared to 27.0% for PCI (*p* < 0.001). The study also observed an increase in coronary revascularization rates for MI and CS over time, from 51.5% in 2002 to 67.4% in 2014 (*p*-for-trend < 0.001). These findings suggest a potential benefit of CABG over PCI in patients with MI and CS, warranting further investigation through randomized trials.

To address this uncertainty, investigators at NYU Langone have proposed the CABG-SHOCK trial [42], a randomized study that would compare culprit-only PCI with staged revascularization against immediate CABG in patients with acute myocardial infarction and CS. The trial’s goal is to determine whether complete surgical revascularization can reduce residual coronary disease and improve survival compared with a PCI-based strategy. However, the study has not yet progressed beyond the planning phase.

## 4. Pre-Shock State: Recognition and Management

Hypotension and CS should be understood as existing along a continuum rather than as distinct conditions. Patients may present in a “pre-shock” state, wherein compensatory mechanisms are beginning to fail, yet overt shock has not yet manifested. This phase is characterized by borderline hypotension (systolic blood pressure typically ranging from 80 to 100 mmHg), elevated lactate levels, and early indicators of tissue hypoperfusion, such as oliguria, tachycardia, cool extremities, and subtle alterations in mental status. Importantly, these symptoms occur in the absence of the sustained severe hypotension and multiorgan dysfunction that are characteristic of classic CS [4].

The fundamental pathophysiology is characterized by an imbalance between cardiac output and systemic demand. Acute ischemic injury, elevated filling pressures, and impaired ventricular–arterial coupling diminish effective forward flow. Even slight reductions in arterial pressure can significantly compromise coronary perfusion in the context of severe coronary disease, triggering a downward spiral of worsening ischemia and ventricular dysfunction [4,34].

Recognizing the onset of pre-shock necessitates vigilance, as the condition may deteriorate rapidly. Laboratory indicators, such as a lactate level exceeding 2 mmol/L or an increasing lactate trajectory, serve as early evidence of inadequate perfusion. Invasive hemodynamic assessments may reveal a cardiac index ranging from 1.8 to 2.2 L/min/m^2^, accompanied by elevated pulmonary capillary wedge pressure, indicating limited cardiac reserve. Clinical warning signs at the bedside include narrowing pulse pressure, increasing vasopressor requirements, and a poor response to fluid challenges. The Society for Cardiovascular Angiography and Interventions (SCAI) classification identifies this phase as Stage B (“Beginning shock”), underscoring its prognostic significance and the necessity for early intervention [4].

Management strategies focus on preventing progression toward established shock. Rapid revascularization of the culprit lesion in the context of ACS is fundamental to therapy, as ongoing ischemia exacerbates hemodynamic instability. Hemodynamic support necessitates precise titration: cautious fluid resuscitation may benefit preload-dependent patients, whereas excessive resuscitation poses a risk of pulmonary edema. Inotropes and vasopressors may offer temporary stabilization; however, escalating requirements often indicate impending deterioration. Structured monitoring—encompassing continuous arterial pressure, serial lactate measurements, and urine output surveillance—is crucial for guiding therapy and detecting deterioration [44].

## 5. Mechanical Circulatory Support in Cardiogenic Shock

### 5.1. Rationale and Pathophysiologic Targets

**Figure 1:**
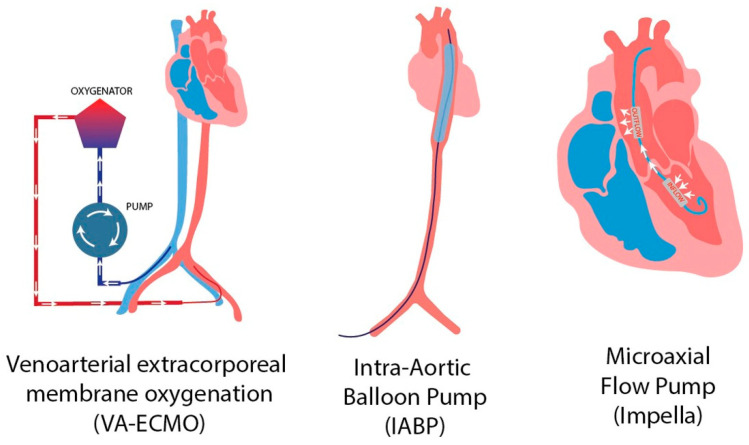
Mechanical circulatory support devices [55]. Reproduced from Putowski Z. et al. J Cardiothorac Vasc Anesth. 2023;37(10):2065-72. CC BY 4.0.

Mechanical circulatory support (MCS) devices play a crucial role in managing CS. While pharmacologic interventions such as vasopressors and inotropes can improve perfusion pressure, they also increase myocardial oxygen demand [30]. In contrast, MCS devices aim to restore systemic blood flow and, depending on the specific platform, reduce ventricular load and wall stress, (Figure 1). This approach creates favorable physiological conditions for myocardial recovery while allowing for definitive therapeutic interventions, such as culprit-lesion revascularization or correction of mechanical complications [31,54].

The selection of an appropriate MCS device is based on several factors, including the patient’s hemodynamic phenotype (left-sided versus biventricular failure, presence of severe hypoxemia), disease trajectory (as assessed by the Society for Cardiovascular Angiography and Interventions [SCAI] staging system), and the institution’s expertise, (Table 3). Contemporary management strategies incorporate SCAI staging and multidisciplinary “shock team” approaches, providing a standardized framework for the escalation and de-escalation of mechanical support [4,30,54].

### 5.2. Intra-Aortic Balloon Pump (IABP)

The use of an IABP increases diastolic coronary pressure and slightly decreases afterload, but it offers limited forward flow support, providing less than 1 L/min. The significant IABP-SHOCK II randomized trial [33], which involved 600 patients with AMI-CS scheduled for early revascularization, found no reduction in 30-day mortality when comparing routine IABP use to no IABP (39.7% vs. 41.3%; RR 0.96, 95% CI 0.79–1.17). There were also no benefits in secondary outcomes or ICU course metrics, with similar rates of bleeding, sepsis, stroke, and limb ischemia in both groups. The results at twelve months remained unchanged. Consequently, routine IABP use in AMI-CS does not enhance survival and is not advised when early revascularization is possible. This evidence supports the downgrade of IABP for STEMI-CS in the 2025 ACC/AHA ACS guideline [1].

### 5.3. Impella

The Impella series consists of percutaneous, transvalvular micro-axial devices designed to assist the left ventricle by drawing blood from it and pumping it into the ascending aorta [57]. This process enhances systemic circulation and reduces the load on the left ventricle, which decreases LVEDP and wall stress, potentially improving coronary perfusion pressure and minimizing ischemic damage in cases of CS [58]. The Impella CP, the most frequently utilized model in AMI-CS, is typically inserted retrogradely through a 14 Fr femoral arterial sheath, crossing the aortic valve under fluoroscopic or echocardiographic guidance [58,59]. When properly positioned and maintained with continuous anticoagulation, it can provide approximately 3.5–4.0 L/min of forward flow. Other models, such as the smaller Impella 2.5 and the surgical/axillary platforms (5.0/5.5), offer varying flow rates, with the latter providing higher outputs. Risks associated with these devices include significant bleeding, vascular injury or limb ischemia, and hemolysis, which require careful management of access, anticoagulation, and monitoring protocols [44,60].

The initial hemodynamic randomized controlled trial, ISAR-SHOCK [61], involving 26 patients with acute myocardial infarction complicated by AMI-CS, compared the Impella 2.5 device to intra-aortic balloon pump (IABP), (Table 2). The study revealed significant acute improvements in cardiac index with Impella, but no difference in 30-day mortality. However, the trial’s small sample size limited its statistical power for clinical outcomes.

Subsequently, the IMPRESS trial [46], which included 48 patients with severe AMI-CS, mostly post-cardiac arrest, compared Impella CP to IABP. The results showed comparable mortality rates at 30 days and 6 months, with no significant difference at 5-year follow-up. However, Impella use was associated with higher rates of bleeding and hemolysis in the short term.

The recent DanGer Shock trial [50], published in the *New England Journal of Medicine* in 2024, represents the first adequately powered randomized controlled trial evaluating Impella in AMI-CS patients undergoing emergency revascularization. This study randomized 355 participants to Impella CP plus standard care versus standard care alone. At 180 days, all-cause mortality was significantly reduced in the Impella group (45.8% vs. 58.5%; hazard ratio [HR] 0.74; 95% confidence interval [CI] 0.55–0.99; *p* = 0.04). However, this survival benefit was accompanied by higher complication rates, including severe bleeding (24.0% vs. 12.8%)**,** vascular complications (19.2% vs. 5.1%), and a greater need for renal replacement therapy (40.6% vs. 26.4%)**.**

These findings necessitate a careful evaluation of the risk-benefit ratio and emphasize the importance of implementing precise implantation and management protocols. The 2025 ACC/AHA ACS guideline [1] reflects these results by providing a Class IIa recommendation for microaxial flow pumps (Impella CP) in select patients with severe or refractory STEMI-CS, while emphasizing the need for caution in their application.

Impella is being assessed in situations of non-CS, as demonstrated in the UK multicenter CHIP-BCIS3 trial [62]. This study involves randomizing patients who have extensive multivessel coronary disease and severe left ventricular (LV) dysfunction, and who are undergoing non-emergent, complex PCI, to either elective percutaneous LV unloading or standard care. The primary endpoint, structured hierarchically as a win-ratio, encompasses all-cause mortality, stroke, spontaneous myocardial infarction (MI), cardiovascular hospitalization, and periprocedural MI, along with integrated cost-effectiveness and quality of life (QoL) analyses. Concurrently, the PROTECT IV [63] randomized controlled trial (RCT) is recruiting high-risk PCI patients with reduced left ventricular ejection fraction (LVEF) to compare Impella-supported PCI against unsupported PCI, with a focus on a composite outcome of death, stroke, MI, or cardiovascular hospitalization over an extended follow-up period. Collectively, these trials aim to determine if prophylactic microaxial support can facilitate more comprehensive revascularization and safer multivessel intervention in cases of advanced LV dysfunction—an area where previous randomized studies, such as PROTECT II [64], indicated hemodynamic benefits without a clear early clinical advantage, and where current guidelines emphasize ongoing uncertainty outside of shock scenarios.

### 5.4. Veno-Arterial Extracorporeal Membrane Oxygenation (VA-ECMO)

VA-ECMO is an advanced mechanical circulatory support system used in severe cardiogenic shock. It temporarily assumes the role of the heart and lungs, providing circulatory and respiratory support through a system of cannulas, an extracorporeal circuit, a pump, an oxygenator, and a heat exchanger. The system drains deoxygenated blood from the venous system, oxygenates it, and returns it to the arterial system, improving systemic perfusion and oxygen delivery to end organs [52]. Recent randomized controlled trials have examined the efficacy of VA-ECMO in CS. The ECMO-CS trial [52] (*n* = 117) randomized patients with severe CS to immediate VA-ECMO versus an initially conservative strategy with rescue ECMO as needed, showing no difference in the composite endpoint of death, resuscitated circulatory arrest, or crossover to urgent ECMO at 30 days (63% vs. 71%; *p* = 0.78). The ECLS-SHOCK trial [51], the largest ECMO RCT to date (*n* = 420 AMI-CS patients), demonstrated 30-day all-cause mortality rates of 47.8% with early ECLS versus 49.0% with standard care (relative risk [RR] 0.98; 95% CI 0.80–1.19; *p* = 0.81). The primary endpoint of 30-day all-cause mortality was not significantly reduced. Importantly, the trial design permitted rescue ECMO in patients who deteriorated, which limits over-interpretation of the conclusion that routine early ECMO is not superior to standard care. The smaller EURO SHOCK pilot trial [56] (*n* = 35) observed a 30-day mortality of 43% in the ECMO arm versus 77% in controls, but the difference did not reach statistical significance (*p* = 0.18) given its limited power. Collectively, these randomized studies indicate that routine early VA-ECMO does not improve short-term survival in AMI-CS and may increase complications. However, selective ECMO use—for example, in patients with profound hypoxemia or refractory biventricular failure—remains a potential consideration.

## 6. Conclusions

Circulatory disturbances in patients with acute coronary syndrome undergoing percutaneous coronary intervention remain a significant challenge in interventional cardiology. This review has highlighted the complex interplay of factors contributing to hemodynamic instability, ranging from vasovagal reflexes and allergic reactions to severe complications like CS. The importance of early recognition and appropriate management of these disturbances cannot be overstated, as they can significantly impact patient outcomes.

The evolution of mechanical circulatory support devices has provided new options for managing severe circulatory compromise, particularly in cases of CS. However, recent randomized controlled trials have yielded mixed results regarding their efficacy. While the DanGer Shock trial demonstrated a mortality benefit with routine Impella CP use in AMI-CS patients, this came at the cost of increased complications. Similarly, studies on VA-ECMO have not shown clear mortality benefits in CS, despite its theoretical advantages.

These findings underscore the need for a nuanced, patient-centered approach to managing circulatory disturbances in ACS patients undergoing PCI. The use of risk stratification tools, such as the SCAI classification system, and the implementation of multidisciplinary “shock team” approaches may help optimize patient care. Future research should focus on refining patient selection criteria for mechanical support devices and developing strategies to mitigate their associated complications.

Ultimately, improving outcomes in this high-risk patient population will require a combination of early recognition, rapid intervention, judicious use of advanced supportive technologies, and ongoing refinement of management protocols based on emerging evidence. As our understanding of the pathophysiology of circulatory disturbances in ACS continues to evolve, so too must our approaches to their prevention and management.

## Figures and Tables

**Table 1:** SCAI Classification of Cardiogenic Shock [4,34].

Stage	Label	Clinical Features	Hemodynamics/Biomarkers	Prognosis/Notes
A	At risk	Patients at risk for shock but without current evidence of hypoperfusion (e.g., large anterior MI, severe valvular disease).	Normal BP, lactate, perfusion; stable hemodynamics.	Lowest risk; early recognition important.
B	Beginning	Early signs of hemodynamic instability; borderline hypotension or rising lactate, but without hypoperfusion.	SBP 90–100 mmHg, narrow pulse pressure, rising lactate (>2 mmol/L).	“Pre-shock” phase; early intervention may prevent progression.
C	Classic	Hypotension with evidence of hypoperfusion (cold extremities, oliguria, altered mentation).	SBP < 90 mmHg or MAP < 60 mmHg, elevated filling pressures, cardiac index ≤2.2 L/min/m^2^, lactate elevated.	Typical shock; requires urgent intervention, inotropes, or MCS.
D	Deteriorating	Failure to stabilize despite initial therapy (inotropes, fluids, vasopressors).	Worsening hemodynamics, escalating support, rising lactate.	Poor prognosis; signals need for escalation (Impella, VA-ECMO).
E	Extremis	Profound shock, often with ongoing CPR, refractory to maximal therapy.	Severe acidosis, hyperlactatemia, minimal or absent cardiac output.	Highest mortality; salvage therapies or palliation considered.

**Table 2:** Summary of Landmark Clinical Trials in Cardiogenic Shock: Revascularization Strategies and Mechanical Circulatory Support.

Study/Source	Design	Population	Strategy	Key Outcomes
SHOCK Trial (NEJM 1999; JAMA 2006), [35,36]	Multicenter RCT, AMI-CS (*n* ≈ 300)	AMI with CS within 36 h, eligible for PCI or CABG	Early revascularization (PCI/CABG) vs. initial medical stabilization	No 30-day mortality reduction, but significant survival benefit at 6–12 mo and sustained long-term
CULPRIT-SHOCK (NEJM 2017; Circulation 2018), [38,39]	Multicenter RCT, *n* = 706	AMI-CS with multivessel disease	Culprit-only PCI (with staged PCI as needed) vs. immediate multivessel PCI	30d death/RRT lower with culprit-only (45.9% vs. 55.4%). At 1 yr: no mortality diff, but higher rehosp/revascularization with culprit-only
Meta-analysis (2018, EuroIntervention/Heart), [43]	Systematic review & meta-analysis of RCT + registry data	AMI-CS with multivessel CAD	Culprit-only vs immediate complete revascularization	Culprit-only safer at index; staged PCI after stabilization reasonable
Observational CABG vs. PCI (Am Heart J 2020; others), [42]	Retrospective registry comparisons	AMI-CS with LM or complex 3-vessel CAD	Primary CABG vs. PCI	CABG associated with lower in-hospital mortality (confounded); no RCT evidence
Guidelines (ESC 2023 ACS; ACC/AHA 2025 ACS), [1,2]	Consensus guidelines	AMI-CS	Culprit-only PCI urgent; avoid routine non-culprit PCI; staged PCI after stabilization; CABG in LM/complex	Class I: culprit PCI; Class III (ACC/AHA): avoid routine complete revasc in shock
NCSI/Shock team protocols, [44]	Observational, system-based care	AMI-CS in networked care models	Early PCI with structured use of temporary MCS + shock team	Improved survival compared with historical cohorts; non-randomized
IABP-SHOCK II (NEJM 2012; 6-yr Circulation 2019), [33,45]	Multicenter RCT, *n* = 600	AMI-related cardiogenic shock planned for early revascularization	Routine IABP vs. no IABP (guideline-directed care incl. PCI/CABG)	No reduction in 30-day mortality; no difference at 12 months or 6 years; supports avoiding routine IABP use
IMPRESS in Severe Shock (Lancet 2017; 5-yr follow-up 2021), [46,47]	Open-label RCT, *n* = 48	Severe AMI-CS undergoing primary PCI	Impella CP vs. IABP	No mortality difference at 30 days or long-term (5 years); higher device-related complications with Impella in small sample
TandemHeart vs IABP (Thiele 2005; Burkhoff 2006), [48,49]	Two RCTs (single- & multicenter), *n* ≈ 41 and *n* ≈ 42	Cardiogenic shock (≈70% AMI) within 24 h; many undergoing PCI	TandemHeart pVAD vs. IABP	Greater hemodynamic improvement with TandemHeart; no 30-day survival benefit; more bleeding/vascular complications
DanGer Shock (NEJM 2024), [50]	Multicenter RCT, *n* = 355	STEMI-related cardiogenic shock	Impella CP + standard care (pre/during/≤12 h post cath) vs. standard care alone	Lower 180-day all-cause mortality with Impella CP (HR ≈ 0.74; *p* ≈ 0.04); higher major bleeding/limb ischemia/hemolysis
ECLS-SHOCK (NEJM 2023), [51]	Multicenter RCT, *n* = 420	AMI-related cardiogenic shock after PCI or during MI care	Early routine VA-ECMO + usual care vs. usual care alone (with rescue ECMO allowed)	No reduction in 30-day mortality (~48–49% both groups); more bleeding and vascular complications with ECMO
ECMO-CS (Circulation 2023; 1-yr Eur J Heart Fail 2025), [52,53]	Multicenter RCT, *n* = 117	Rapidly deteriorating or severe cardiogenic shock (majority AMI)	Immediate VA-ECMO vs. early conservative care (bailout ECMO allowed)	Primary composite at 30 days not reduced (HR ≈ 0.72; *p* = 0.21); no difference in mortality; neutral 1-yr outcomes; safety concerns similar

**Table 3:** Comparative Features of MCS Devices.

Device	Flow Support	Hemodynamic Effects	Advantages	Limitations	Best Suited For
IABP	<1 L/min	↑ Diastolic pressure, mild ↓ afterload	Easy insertion, few complications	Minimal support, no mortality benefit	Mild LV failure, bridge in centers lacking advanced MCS
Impella CP	3.5–4 L/min	Direct LV unloading, ↑ CO	Improved hemodynamics, RCT mortality benefit (DanGer-shock)	Bleeding, hemolysis, vascular injury	STEMI-CS with severe LV failure, high-risk PCI
VA-ECMO	4–6 L/min (full CP support)	Biventricular + oxygenation	Life-saving in refractory arrest/hypoxemia	↑ Afterload, bleeding, limb ischemia, no RCT mortality benefit	Profound shock, cardiac arrest, severe hypoxemia

↑: augmentation; ↓: reduction; Data derived from pivotal randomized trials including IABP-SHOCK II [33], DanGer Shock [50], ECLS-SHOCK [51], ECMO-CS [52], and EURO SHOCK [56], together with guideline recommendations [1,2].

## Data Availability

No new data were created or analyzed in this study. Data sharing is not applicable to this article.
